# Simple and Low-Cost Footstep Energy-Recover Barocaloric Heating and Cooling Device

**DOI:** 10.3390/ma14205947

**Published:** 2021-10-10

**Authors:** Javier Garcia-Ben, Ignacio Delgado-Ferreiro, Jorge Salgado-Beceiro, Juan Manuel Bermudez-Garcia

**Affiliations:** Department of Chemistry, Faculty of Science and Advanced Scientific Research Center (CICA), University of A Coruna, QuiMolMat Group, Zapateira, 15071 A Coruña, Spain; javier.garcia.ben@udc.es (J.G.-B.); ignacio.delgado.ferreiro@udc.es (I.D.-F.); jorge.salgado@udc.es (J.S.-B.)

**Keywords:** barocaloric device, eco-friendly refrigeration, energy recovery

## Abstract

In this work, we design, build, and test one of the very first barocaloric devices. The here presented device can recover the energy generated by an individual’s footstep and transform it into barocaloric heating and/or cooling. Accordingly, we present an innovative device that can provide eco-friendly and gas-free heating/cooling. Moreover, we test the device by measuring a new barocaloric organic polymer that exhibits a large adiabatic temperature change of ~2.9 K under the application of 380 bar. These results pave the way towards novel and more advanced barocaloric technologies and provide a simple and low-cost device to explore new barocaloric materials.

## 1. Introduction

In recent years, solid-state barocaloric materials have arisen as a promising solution for gas-free and eco-friendly refrigeration systems [1,2,3,4]. Barocaloric materials are solid-state compounds that exhibit large thermal changes, meaning isothermal entropy changes (Δ*S*) or adiabatic temperature changes (Δ*S*), defined as barocaloric effects. These thermal changes are generally related to volume changes (Δ*v*) in the materials induced by the application of external pressure. In that way, the volume changes can be related to first-order phase transitions where the barocaloric materials display structural transformations between different polymorphs. On the other hand, barocaloric effects can also arise from the pressure-induced volumetric compression and expansion of the aforementioned barocaloric materials without experiencing any structural transition.

In this context, it should be noted that most conventional refrigeration systems still rely on the vapor compression (and expansion) of gas refrigerants, mainly hydrofluorocarbons (HFCs), hydrocarbons (HCs), ammonia, and/or CO_2_. Nevertheless, all these gases show important drawbacks. For instance, HFCs exhibit very large global warming potential (GWP), sometimes thousands of times larger than CO_2_. For that reason, the International Kigali Agreement aims to remove 80% of those fluorinated refrigerants by the year 2030. Trying to overcome these critical inconveniences, HCs, ammonia, and CO_2_ offer very low (almost negligible) GWPs. However, these gases are flammable, corrosive, toxic, and/or asphyxiating compounds, which also pose important risks for the user.

In comparison with gas refrigerants, barocaloric materials are eco-friendlier, while also being easier and safer to transport and manipulate. To further elaborate, solid barocalorics cannot escape to the atmosphere (avoiding direct greenhouse gas emissions), they can be easily recovered and reused in case of an accidental leak, they offer higher compactness (saving installation and transport space), they can be transported in non-pressurized containers (avoiding explosion risks), and they are not flammable and/or asphyxiating compounds.

In view of these advantages, in the last years many efforts have been devoted to the development and study of new barocaloric materials. As a consequence, giant (Δ*S* > 10 J K^−1^ kg^−1^, Δ*T* > 5 K) and even colossal (Δ*S* > 100 J K^−1^ kg^−1^, Δ*T* > 20 K) barocaloric effects have been found in different families of materials, such as metallic alloys [5,6,7,8], inorganic ammonium salts [9,10,11], organic plastic crystals [12,13,14,15], superionic conductors [16,17], spin-crossovers [18,19,20], hybrid organic-inorganic compounds [21,22,23,24], and organic polymers [25,26,27,28,29,30].

However, despite all the advances in the exploration of new barocaloric materials, fewer efforts were devoted to the development of new devices and/or prototypes that can measure the performance and/or provide cooling using barocaloric compounds [4,31,32]. In that regard, the barocaloric performance of the aforementioned barocaloric materials were mainly studied by calculating the isothermal entropy change from differential scanning calorimetry with coupled pressure generators (gas and/or oil pressurizing pumps). Only a few barocaloric materials, and especially the barocaloric organic polymers, were studied by thermometry under pressure. Here, thermocouples were used to register the temperature change, and a high-pressure hydraulic press (*p* = 260–3900 bar) was used to apply the pressure to the materials inserted in a stainless steel vessel [29]. Therefore, barocaloric thermometry devices (that can measure adiabatic temperature changes) are even scarcer than high-pressure differential scanning calorimeters (that can analyze isothermal entropy changes). Up-to-date, both types of devices require a large work input from the hydraulic pumps/press to provide large pressures, generally of above 1000 bar.

In this work, we develop a new barocaloric thermometry device (from now on barocaloric scale) that recovers the work from footsteps to provide the pressure required to induce a heating/cooling effect with solid-state barocaloric materials. We anticipate that footstep-induced cooling and/or heating devices could be implemented in future technologies, including running shoes and or buildings’ floors, to maintain a comfortable temperature by recovering the energy of our own body locomotion. Even though important engineering challenges must be addressed before practical implementation, such as the design of adequate heat sinks (for instance, serpentines with heat-transfer fluids) that can conduct the heat towards/from the desired space. Another important challenge will be optimizing the amount of the barocaloric material and the size of the barocaloric system to adapt to large spaces such as building floors and to confined spaces such as shoes.

In this context, energy harvesting wearables were recently explored in shoes and/or insoles to transform human mechanical energy into electrical energy [33,34,35]. However, the here presented device is the first one (to our knowledge) that can transform human movement into cooling and/or heating. In order to test this device, we also measured, for the first time, the adiabatic temperature change of the organic polymer trans-1,4-polyisoprene, and we compared it with the reported barocaloric performance of the vulcanized natural rubber (mainly cis-1,4-polyisoprene subjected to a Sulphur vulcanization process) [29].

## 2. Design, Components, and Dimensions of the Barocaloric Scale

**Figure 1:**
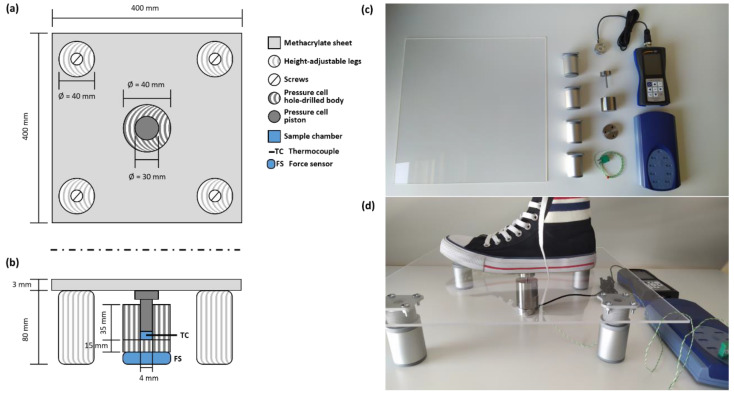
Schematics of the top (**a**) and lateral (**b**) views of the barocaloric scale with the dimensions of its components, and photographs of the actual components (**c**) and final assembling (**d**) of the barocaloric scale.

The barocaloric scale has been designed with low-cost and, mainly, off-the-shelf components (see Figure 1). The structural part consists of a methacrylate sheet (400 × 400 × 3 mm) and four height-adjustable legs (height: 60–80 mm) screwed on the corners of the sheet. The pressure-cell containing the barocaloric material is a homemade stainless-steel vessel consisting of a hole-drilled body and a piston (see Figure 1), which is located in the middle of the methacrylate sheet. The cell body is divided into two parts: (1) a stainless-steel solid base of 40 mm of diameter and 15 mm of height that is screwed to (2) a hole-drilled cylinder of 40 mm of diameter and 35 mm of height, where the drilled hole is situated in the middle of the cylinder with a diameter of 4 mm and a height of 35 mm. Here, the sample (around 10 mg) is placed inside the drilled hole on top of the solid base (see Figure 1b). A piston of 4 mm of diameter and 35 mm of height is placed inside the drilled hole on top of the sample. This piston counts with a wider head of 300 mm of diameter and 10 mm of height to facilitate the contact with the methacrylate sheet and the force transmission of the footstep. The force of the footstep is measured using a 2 kN button-shaped compression load cell connected to a PCE-DFG N dynamometer (PCE Iberica, Tobarra, Spain) and placed underneath the homemade pressure cell. The temperature of the sample was monitored using a K-type thermocouple of 0.2 mm of diameter connected to a Picolog TC-08 data logger (Pico Technology, St. Neots, UK). The thermocouple was placed in the middle of the sample through a wall bushing located in the side of the homemade pressure cell (see Figure 1b). Figure 1c,d show photographs of the actual elements and device assembling used for the barocaloric scale. As previously mentioned, this barocaloric device was simple and inexpensive with a total cost (including all the components, the homemade pressure cell, the dynamometer, and the temperature data logger) of 1700 €, which can be easily within the budget of the most modest academic and/or school laboratories.

**Figure 2:**
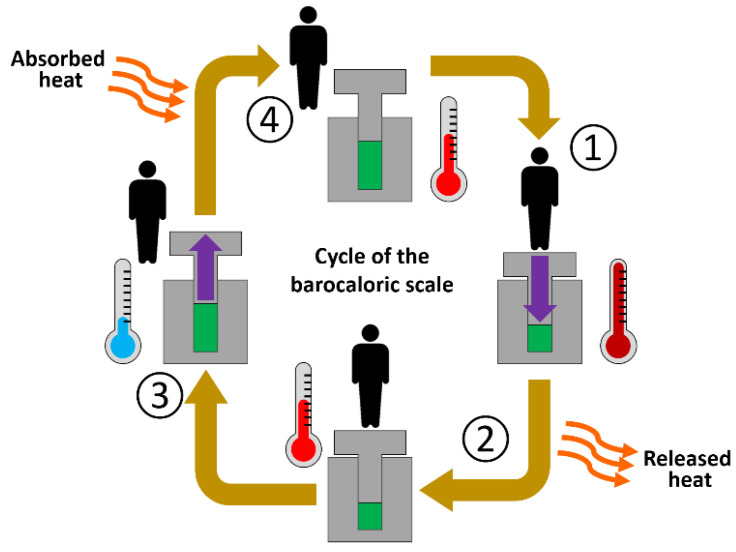
Simple schematics of a barocaloric heating/cooling cycle generated in our barocaloric scale.

The barocaloric scale is designed to study the barocaloric cycle of pressure-induced heating and cooling by using the force of the footstep as pressure source. In a simple and general way, a conventional barocaloric cycle consists of the following four steps (see Figure 2): (1) when pressure is applied to a barocaloric material, the material increases its temperature, (2) by maintaining the applied pressure, the barocaloric material releases the generated heat, and its temperature decreases back to its initial state, (3) by releasing the applied pressure, the barocaloric material further decreases its temperature below the initial state, and (4) by maintaining the pressure released, the material absorbs heat from the environment and increases its temperature up to its initial value. Here, the released heat could be used to warm-up a given space in a heating process, and/or the heat absorption could be used to remove heat from a given place in a cooling process [36].

## 3. Measurement of the Temperature Changes Induced by Footstep in Trans-1,4-polyisoprene

To test our barocaloric scale, we used the organic polymer trans-1,4-polyisoprene as potential barocaloric material, whose barocaloric effect was not previously measured. However, we anticipated its potential barocaloric performance by analogy with the previously reported barocaloric vulcanized natural rubber, which was formed mostly by cis-1,4-polyisoprene processed through sulfur vulcanization [29]. We used as-purchased commercial trans-1,4-polyisoprene instead of commercial cis-1,4-polyisoprene because the first one was solid at room temperature meanwhile the second was a viscous liquid in the same conditions. Furthermore, the material was inexpensive (~0.2 €/kg) and can be easily manipulated and shaped due to its mechanical resistance and thermoplastic properties with thermal stability up to ~330 K [37].

We inserted around 10 mg of the sample inside the homemade pressure cell, and we measured the adiabatic temperature changes of the organic polymer at room temperature (*T* = ~300 K). It should be noted that the homemade pressure cell was designed to work at ambient temperature and, therefore, the operating temperature cannot be tuned. For applying the required force, we selected three individuals with different weights (61.8 kg, 71.9 kg, and 85.0 kg, respectively) to repeatedly step on and off our barocaloric scale.

**Figure 3:**
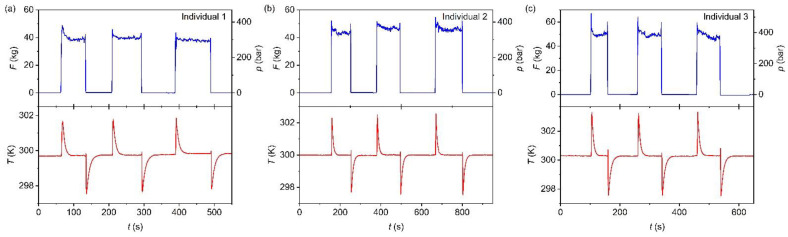
Selected curves of force and pressure (blue curves) and temperature (red curves) as a function of the cyclic footsteps of individual (**a**) 1, (**b**) 2, and (**c**) 3 with a bodyweight of 61.8 kg, 71.9 kg, and 85.0 kg, respectively.

The footstep of the individuals on top of the scale generated a registered force of between ~37.2 and ~48.7 kg that, considering the dimensions of the piston, corresponds to an applied pressure of between ~290 and ~380 bar. As can be observed in Figure 3, when applying the footstep pressure, the temperature of the trans-1,4-polyisoprene increased, meanwhile when the footstep pressure was removed, the temperature of the material decreased. This was consistent with the behavior of a conventional barocaloric material [38]. The here tested barocaloric material and the device shows a good preliminary cyclability along the experiment, where the loading cycles were performed on the same sample over time without affecting its barocaloric response. In that regard, further long-term cyclability studies must be performed to evaluate the stress fatigue of the devices and barocaloric materials before any commercial implementation. However, those specific and long-time consuming experiments were out of the scope of the present work.

In addition, we also observed that the barocaloric response was very fast: the temperature reached its maximum/minimum value in ~2 s after applying/removing the footstep force. Meanwhile, the material’s thermalization was a bit slower, needing around ~35 s to reach room temperature back. This relatively long thermalization time could be related to the very low thermal conductivity of polyisoprene, which shows a value of λ ~0.14 W m^−1^ K^−1^ [39]. It should be noted that the fast pressure-induced temperature change and the low thermal conductivity of the sample facilitates a *quasi*-adiabatic temperature change where the heat transfer from the sample to the environment was almost negligible during the fast pressure application (~2 s long). Later, when more time was given to the system, this heat was transferred to the surroundings (i.e., the pressure-cell made of stainless steel, which was a relatively good thermal conductor), giving rise to the barocaloric material thermalization. It should be noted that, in our current device, the ratio between cell-mass and sample-mass was very large, and, therefore, the transferred heat will rapidly dissipate across the cell walls without increasing their temperatures significantly. Future advancements will consider the design of adequate heat sinks that can intentionally conduct the heat towards a specific space. However, the present work mainly focuses on demonstrating that it is possible to use human movement to generate thermal changes in barocaloric materials, which will open the doors towards more advanced devices in the future.

**Figure 4:**
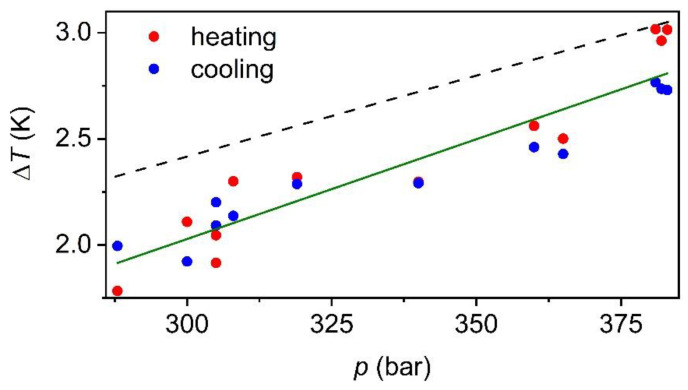
Temperature change (Δ*T*) as a function of the pressure-induced by different footstep forces from 290 to 380 bar. Note: green solid line represents the linear trend of the here obtained experimental data for trans-1,4-polyisoprene. Meanwhile, the black dashed line represents the thermodynamic model reported for the isomer compound cis-1,4-polyisoprene [40].

Furthermore, we also observed that the larger was the applied pressure, the larger was as well the temperature change of the material (see Figure 4). Actually, the barocaloric response of the material seems to follow a linear behavior from ~1.9 K up to ~2.9 K when applying a footstep pressure from ~290 bar to ~380 bar, according to equation $\Delta T=-0.8+9.4\times 10^{-4}\times p$ (see Figure 4).

In this regard, we found that this material exhibited a similar barocaloric effect to the reported barocaloric vulcanized natural rubber, whose main component was sulfured cis-1,4-polyisoprene, and that exhibited a barocaloric effect of ~3.0 K when applying a slightly larger pressure of 434 bar [29]. Moreover, these values were in agreement, showing slight Δ*T* differences, with the theoretical calculations obtained by molecular dynamics and thermodynamics modeling as observed in Figure 4 [40]. Additionally, such computational studies revealed that the barocaloric effects in polyisoprene were related to a polymer chains rearrangement upon compression, which led to a reduction in its free volume and chain mobility. Therefore, these results validated the adequate operation of our barocaloric scale.

On the other hand, it should be noted that, according to the recent barocaloric technology roadmap of the Henry Royce Institute [41], barocaloric materials should operate under pressures up to 300 bar in order to provide efficient and low-energy consumption refrigeration technologies. In this case, we found that trans-1,4-polyisoprene operated under pressures near 300 bar. Moreover, the designed barocaloric device did not require the production of external energy but recovered and reused the energy of the body movement through our footsteps, which could pave the way towards more efficient cooling systems. This was especially important since the same roadmap identified that one of the main challenges in barocaloric technologies was to explore alternative mechanisms to apply the required pressure in a practical manner.

Furthermore, although a temperature change of around ~3 K was clearly not sufficient to provide cooling for a fridge, freezer, and/or air-conditioner, it could be well enough to optimize our body’s thermal comfort. As it is well known, a change of a few decimals in the human body’s temperature can dramatically affect our comfort and performance. This is observed, for instance, when athletes are competing under hot thermal stress [42]. Actually, there are many professionals suffering from thermal stress that could benefit from self-refrigerated clothing and PPEs. That is the case, for instance, of firefighters, miners, welders, construction, and factory workers, and even healthcare workers, as evidenced by the COVID-19 pandemic [43]. Therefore, in these situations, a cooling down of a couple of temperature degrees would be sufficient to improve our body comfort and performance.

## 4. Conclusions

In this work, we have designed an innovative and low-cost barocaloric device that recovers the energy of individuals’ footsteps and transforms it into heating and cooling. This device is the first demonstrator that highlights new possibilities of using human motion to provide gas-free and eco-friendly heating and cooling, which could be implemented in future wearables technologies (such as shoes or insoles and/or self-refrigerating floor buildings). Moreover, we have tested this device using the barocaloric organic polymer trans-1,4-polyisoprene, whose barocaloric effect was not previously reported and is confirmed by our work. In that regard, this new barocaloric material exhibits an adiabatic temperature change of up to 2.9 K when applying footstep pressures of up to 380 bar, near the operating pressure recommended by the last barocaloric technology roadmap of the Henry Royce Institute. In addition, we have found that the barocaloric response of the here studied compound is similar to other organic polymers, such as vulcanized natural rubber. Overall, this work opens the door towards future and more advanced energy-recovery heating and cooling technologies. This work also provides a simple and low-cost testing barocaloric device for academic research and school teaching projects, as well as a science communication device that has already been used in long-term museums exhibitions and national educational TV programs [44,45].

## Data Availability

All data are available from the authors by request.
